# Receptive and participatory arts engagement and subsequent healthy aging: Evidence from the Health and Retirement Study

**DOI:** 10.1016/j.socscimed.2023.116198

**Published:** 2023-10

**Authors:** Melinda Rena, Daisy Fancourt, Feifei Bu, Elise Paul, Jill K. Sonke, Jessica K. Bone

**Affiliations:** a Research Department of Behavioural Science and Health, Institute of Epidemiology and Health Care, University College London, London, UK; b Center for Arts in Medicine, University of Florida, Gainesville, FL, USA

**Keywords:** Cultural engagement, Chronic disease, Cognition, Physical functioning, Mental health

## Abstract

**Rationale:**

Arts engagement is associated with prolonged longevity, but it remains unclear whether it is also associated with increases in the portion of people's lives for which they remain healthy. We investigated whether receptive and participatory arts engagement were associated with healthy aging two and four years later.

**Method:**

We included 1269 older adults from the Health and Retirement Study (HRS), a longitudinal study of individuals aged 50 and above in the United States. Participants who completed the HRS 2014 Culture and the Arts Module and who were alive in 2016 and 2018 were eligible. We measured the number of participatory arts activities engaged in (e.g., crafts, dancing) and frequency of receptive arts engagement (e.g., going to a gallery or performance) in the past year. Healthy aging was a binary outcome, conceptualized as no major chronic diseases, no cognitive impairment, good physical functioning, and good mental health.

**Results:**

In logistic regression models, doing receptive arts once a month or more was associated with higher odds of healthy aging four years later compared to never engaging (odds ratio [OR] = 1.80, 95% CI = 1.10, 2.96). However, this evidence was attenuated after adjusting for demographic and socioeconomic covariates (adjusted OR = 1.44, 95% CI = 0.84, 2.46). The number of participatory arts activities engaged in was not associated with healthy aging two or four years later. In sensitivity analyses, there was some evidence that receptive engagement was associated specifically with higher odds of good physical functioning four years later.

**Conclusions:**

The lack of consistent associations between receptive and participatory arts engagement and healthy aging was unexpected given previous evidence for links between arts engagement and each of the four domains of healthy aging. Our findings highlight key methodological issues that should be explored in further research with larger nationally representative samples, longer follow-ups, and more detailed measures of arts engagement.

## Highlights

• Some evidence that receptive arts engagement supported healthy aging 4 years later
• Association driven by higher odds of good physical functioning 4 years later
• No evidence that participatory arts engagement was associated with healthy aging
• Inconsistent evidence overall highlights methodological issues for further research

## 1 Introduction

Globally, the number of older adults is increasing at a faster pace than all other age groups. For example, in the United States (US), adults aged 65 and above are projected to form 23% of the population by 2060 (Vespa et al., 2020). As the number of older adults increases, identifying ways to maintain healthy aging becomes increasingly important for population-level wellbeing and limiting healthcare costs (World Health Organization, 2020). Although the definition of healthy aging has been subject to debate, Rowe and Kahn's (1987, 1997) multisystem characterization of successful aging is widely used. This definition includes good cognitive and physical functioning, low probability of chronic diseases, and optimal mental and social wellbeing. The World Health Organization's (WHO) definition of healthy aging similarly includes “developing and maintaining the functional ability that enables well-being in older age”, but this is regardless of the presence of illness (World Health Organization, 2020). Other definitions also emphasize the importance of functional ability beyond the mere absence of illness, as chronic conditions are not necessarily associated with poor health if they are well managed (Tinetti and Fried, 2004).

Consequently, it is important that research not only focuses on extending people's lifespan or reducing physical and mental health problems, but also on increasing their likelihood of healthy aging, extending the period for which they remain healthy and can enjoy life (the “healthspan”; Kaeberlein, 2018). This is reflected in the United Nations Sustainable Development Goal to promote health at all stages of life (United Nations, 2015). Staying healthy may be particularly challenging later in life as close social ties may be lost, people are more likely to live alone, many have low income, and progressive age-related chronic diseases may limit activities (World Health Organization, 2020). Although research has focused on risk factors that prevent healthy aging, positive health behaviors are also modifiable. There is increasing interest in referring older adults to engage in community leisure activities to support their health (social prescribing), with particular attention on the arts (Fancourt and Finn, 2019; Fraser et al., 2015). Arts activities could involve a range of health promoting ‘active ingredients’, such as opportunities for creative expression, cognitive stimulation, physical activity, sensory activation, and social interaction (Dunphy et al., 2019; Fancourt and Finn, 2019; Warran et al., 2022). The ingredients may then activate a range of mechanisms that could improve health, including psychological processes (e.g., improving mood), biological processes (e.g., reducing levels of stress hormones), social processes (e.g., reducing loneliness), and behavioral processes (e.g., enhancing motivation to engage in other health behaviors; Fancourt et al., 2021).

Large longitudinal population-based studies have shown that arts engagement is associated with a lower risk of premature mortality in older adults (Bavishi et al., 2016; Fancourt and Steptoe, 2019b; Story et al., 2021), demonstrating a relationship with one's ‘lifespan’. Studies have also shown associations with specific components of healthy aging, suggesting a potential relationship with one's overall ‘healthspan’. For example, there has been extensive research on the potential benefits of arts engagement for older adults' mental health, including reducing depression (Bone et al., 2022; Elsden and Roe, 2020) and enhancing wellbeing (Bone et al., 2023a; Groot et al., 2021; Grossi et al., 2012; Ho et al., 2019; Tymoszuk et al., 2019). There is also evidence that visiting museums, galleries, concerts, and theatres and doing creative activities is associated with reduced risk of cognitive impairment (Fancourt and Steptoe, 2018; Iwasa et al., 2012; Petrovsky et al., 2021; Sugita et al., 2021). However, findings have differed across domains of cognition (Bone et al., 2023b; Wang et al., 2013), and some studies have not found consistent evidence for these associations after accounting for socioeconomic factors (Eriksson Sörman et al., 2014; Sugita et al., 2021).

Fewer studies have examined maintenance of physical functioning and chronic diseases. Although there is evidence that going to museums, galleries, concerts, and theatres (Fancourt and Steptoe, 2019a) and doing cognitive, musical, and art activities (Komatsu et al., 2019) is associated with lower risk of subsequent functional disability, these studies have been limited to those who were healthy at baseline. It remains unclear whether arts engagement can maintain or improve physical functioning for older adults with functional impairment. Intervention studies have found arts interventions can support the management of chronic diseases such as cardiovascular diseases and cancer (Fancourt and Finn, 2019), but there is no epidemiological evidence showing whether everyday arts engagement can prevent or help in the management of these diseases.

Furthermore, previous research on arts engagement in older adults focusses on one specific disorder or outcome. To date, no research has tested whether arts engagement is associated with the overall likelihood of healthy aging, combining each of these component parts. As healthy aging is broad and multidimensional, extending beyond the absence of illness to also include the maintenance of physical functioning, it is important to consider the concept as a whole. Additionally, previous studies generally have not compared different types of arts activities, often focusing on specific activities. Individual activities are subject to personal choice and tastes, thus limiting the generalizability of these findings. Grouping activities provides a broader way of assessing types of engagement involving similar ‘active ingredients’ that may be participated in by a wider demographic. Although arts activities can be categorized in many ways, they are commonly split into receptive activities, involving art that has been created and is now experienced by an audience, and participatory activities, requiring the creation of or participation in the arts (Fancourt and Finn, 2019). Receptive activities include going to a museum, gallery, or theatre whereas participatory activities include painting, making music, and photography. Receptive activities are more likely to involve attending an event or venue than participatory activities, most of which can be done at home. Accordingly, there may be more socioeconomic barriers to receptive than participatory arts engagement (Bone et al., 2021). Comparing the associations between receptive and participatory engagement and healthy aging is also important for older adults who have problems with mobility, arthritis, or other factors that may limit the accessibility of some activities.

In this study, we drew our sample from a large cohort of older adults in the US to explore associations between engagement in receptive and participatory arts activities and subsequent healthy aging. Receptive arts included going to a movie, gallery, arts or crafts fair, or live performance. Participatory arts included reading, crafts, making music, acting, dancing, writing, needlework, woodwork, and visual arts. We aimed to investigate whether both types of arts engagement were protective for healthy aging, conceptualized using a multidimensional definition. We hypothesized that both receptive and participatory arts engagement would be associated with higher odds of healthy aging two and four years later.

## 2 Methods

### 2.1 Sample

Participants were drawn from the Health and Retirement Study (HRS), a nationally representative study of more than 37,000 individuals over the age of 50 in the US (Sonnega et al., 2014). The study was initiated by the National Institute on Aging and conducted by the Institute for Social Research at the University of Michigan to track the Baby Boom generation's transition from work to retirement. The initial HRS cohort was interviewed for the first time in 1992 and followed-up every two years, with other studies and younger cohorts merged with the initial sample. Together, these studies create a fully representative sample of individuals over the age of 50 in the United States. Further details on study design are reported elsewhere (Sonnega et al., 2014). The HRS survey was completed on the telephone (2014 = 44%, 2016 = 45%, 2018 = 32%), face to face (2014 = 56%, 2016 = 55%, 2018 = 56%), or online (2018 = 12%). In this study, we combined six HRS public datasets (HRS, 2018 Tracker Final Release [V1.0], RAND HRS Longitudinal File, 2018 [V1]; RAND HRS Detailed Imputations File, 2018 [V1], 2014 RAND HRS Fat File [V2 B]; 2016 RAND HRS Fat File [V2 B]; 2018 RAND HRS Fat File [E1A]).

HRS participants were eligible for inclusion in our study if they completed the 2014 Culture and the Arts Module (CAM) and were alive for subsequent HRS waves in 2016 and 2018. A random 10% subsample of the core HRS sample were eligible for the CAM. In total, 1496 participants completed the CAM, 1314 (88%) of whom were still alive in 2018. We restricted this sample to participants aged 50 years and older in 2014 (n = 40, 3% excluded) and those with complete data on receptive and participatory arts engagement (n = 5, <1% excluded). This resulted in a final analytical sample of 1269 participants.

### 2.2 Ethical approval

All participants gave informed consent, and this study has approval from the University of Florida (IRB201901792) and University College London Research Ethics Committee (project 18,839/001).

### 2.3 Exposures

#### 2.3.1 Receptive arts

The 2014 CAM included two self-report questions on frequency of receptive arts engagement. Participants were asked one question on whether, in the past 12 months, they had been to a receptive activity (yes, no; any of a movie, art museum or gallery, arts or crafts fair, or a live performance such as a concert, play, or reading). Participants who responded yes to this question were then asked how often they went to these types of events in the past 12 months (less than once a month, one to three times a month, once a week, more than once a week). We combined responses to these two questions to create a measure of the frequency of receptive arts engagement in the last 12 months: never, less than once a month, once a month or more. Very few participants engaged more than once a month, so more frequent engagement could not be examined separately.

#### 2.3.2 Participatory arts

The 2014 CAM also included nine self-report questions on participatory arts engagement. In contrast to receptive arts questions, which asked about the frequency of engagement, participatory arts questions measured the variety of activities engaged in. Participants were asked whether, in the past 12 months, they did: (1) read novels, short stories, poetry, or plays, (2) paint, sculpt, pottery, or ceramics, (3) sing or play a musical instrument, (4) act in theatre or film, (5) dance, (6) write stories, poetry, or plays, (7) weave, crochet, quilt, needlepoint, knitting, sewing, or jewelry, (8) leatherwork, metalwork, or woodwork, and (9) photography, graphic design, or filmmaking. Response options were yes or no. To assess the overall levels of participatory arts engagement, we calculated the total number of participatory arts activities engaged in: none, one activity, two activities, three or more activities. Although there has been debate over whether reading should be classified as participatory or receptive, we included it as a participatory activity. Reading requires many skills involved in making art, including active engagement in choosing what to read, the use of imagination and creativity to bring the content to life, integration with own experiences, and new aesthetic experiences (Cliff Hodges, 2010). Given this debate, we also excluded reading from our definition of participatory engagement in sensitivity analyses.

### 2.4 Outcome

We adapted a multidimensional definition of healthy aging which was previously developed in the HRS sample (Kim et al., 2019). At each wave, participants had to meet four criteria to be classed as healthy: (1) free of major chronic diseases, (2) no cognitive impairment, (3) good physical functioning, and (4) good mental health. Participants were considered healthy agers if they met criteria for being healthy across all four components. Healthy aging was thus a binary outcome (yes, no) at all waves, as well as being tested across each of the four separate domains in sensitivity analyses. An overview all healthy aging composite measures is presented in Table S1. There were weak positive correlations between the four domains of healthy aging (Table S2).

We included chronic diseases that are primary causes of mortality in the US; cancer or malignant tumor of any kind (excluding minor skin cancer), heart disease (including heart attack, coronary heart disease, angina, congestive heart failure, or other heart problems), stroke, diabetes, and chronic lung disease (including chronic bronchitis and emphysema but not asthma). Participants were considered healthy in this domain if they did not have any of these diseases or if they were not actively receiving treatment nor taking medication for them (Kim et al., 2019). All items were self-reported. The validity and reliability of self-reported chronic disease has previously been demonstrated in HRS (Fisher et al., 2005).

Cognitive impairment was assessed using the modified Telephone Interview for Cognitive Status (McCammon et al., 2022; Ofstedal et al., 2005). This included a ten-item immediate and delayed free recall test, a serial sevens subtraction test, and a 20-item backward count test. Total scores ranged from 0 to 27, with lower scores indicating cognitive impairment. A threshold for healthy aging was determined based on previous research (Crimmins et al., 2011; Kim et al., 2019); those scoring 12 or more were classified as healthy in this domain.

Good physical functioning was measured by self-reported limitations in a total of ten activities: four physical functions (pushing or pulling large objects, lifting or carrying objects weighing ten pounds, reaching or extending arms up, and stooping kneeling or crouching) and six activities of daily living (walking across a room, dressing, eating, bathing, getting in/out of bed, and using the toilet). Participants reporting difficulties with less than three activities were considered healthy in this domain. This was reduced from difficulties with less than four activities in the previous definition of healthy aging (Kim et al., 2019) as data on four physical functions (walking several blocks, climbing one flight of stairs, getting up from a chair, sitting for 2 h) were missing in 2018, so these activities were excluded from our definition.

We added the fourth domain of good mental health to the previously validated definition of healthy aging (Kim et al., 2019) because healthy aging definitions often include mental health (Rowe and Kahn, 1987, 1997). It was excluded from the previous study, which tested the association between optimism and healthy aging, because mental health may have been too strongly linked to optimism (Kim et al., 2019). Participants were considered to have good mental health, and therefore classified as mentally “healthy”, if they did not have any emotional, nervous, or psychiatric problems. Participants self-reported whether they had, or a doctor told them that they had, any emotional, nervous, or psychiatric problems at each wave.

### 2.5 Covariates

Covariates were factors that have previously been found to predict engagement in receptive or participatory arts activities (Bone et al., 2021; Mak et al., 2020b), which were also likely to be associated with healthy aging. For example, women and people of White race/ethnicity are more likely to participate in the arts. There is also a social gradient in arts participation, whereby people of higher socioeconomic position, as indicated by educational attainment, employment status, and income, are more likely to engage in these activities (Bone et al., 2021; Mak et al., 2020b). Further, independent of individual demographic and socioeconomic background, participation differs according to neighborhood characteristics such as area deprivation (Mak et al., 2020a).

We therefore included demographic and socioeconomic covariates self-reported in 2014 (baseline). Demographic factors were age (years), gender (men, women), marital status (married [including cohabiting], unmarried), and race/ethnicity (White/Caucasian, Black/African American, Other [including American Indian, Alaskan Native, Asian or Pacific Islander, Hispanic, Other]). In the public HRS data, this indicated the race/ethnicity as which participants primarily identified; detailed information was removed to protect participant confidentiality. Socioeconomic factors were educational attainment (less than high school, high school, college, postgraduate), employment status (employed, not working [unemployed, temporarily laid off, disabled, homemakers], retired), total household income (continuous US dollars), and neighborhood safety (excellent/good, fair/poor).

### 2.6 Statistical analysis

We first explored the sociodemographic characteristics of the sample at baseline. We then investigated whether engagement in receptive and participatory arts activities was associated with subsequent healthy aging. We used logistic regression models to test whether arts engagement at baseline (2014) was associated with healthy aging two and four years later (2016 and 2018). Both receptive and participatory arts engagement were included as exposures in the same model. We adjusted all models for the binary indicator of healthy aging at baseline (2014), as subsequent healthy aging is not only related to arts engagement but also to previous healthy aging. Models thus estimate the associations between arts engagement and change in healthy aging status two and four years later. Models are presented before and after adjustment for covariates. Given that the sample included 32 spousal pairs, and spouses are likely more similar to each other than to other participants, we used cluster-robust standard errors.

We used separate logistic regression models for the outcome in 2016 and 2018, as opposed to modelling healthy aging across all years, to ensure there was clear temporal ordering between the exposure and outcome. This also allowed us to control for the baseline measure of the outcome, accounting for any reverse causality. We aimed to first explore whether associations were present at two years after arts engagement, and then test whether any relationships were maintained over a longer period of four years.

For participants with missing data on healthy aging at any wave or covariates, including those lost to follow-up in 2016 and 2018, we imputed data using multiple imputation by chained equations (MICE; White et al., 2011). We used logistic and ordinal regression and predictive mean matching according to variable type, generating 40 imputed data sets (maximum missing data 39%; Table S3). The imputation model included all variables used in analyses and several auxiliary variables (covariates measured in 2016 and 2018: age, marital status, employment, neighborhood safety). All variables were successfully imputed. Although the complete case sample was smaller (n = 668; Tables S5–S6), the results were similar between complete cases and imputed data (Table S7), so imputed findings are reported. All analyses were performed using Stata 17 (StataCorp, 2021).

#### 2.6.1 Sensitivity analyses

First, we explored the concurrent associations between receptive and participatory arts engagement and healthy aging at baseline. Second, given concerns about whether a binary indicator measures healthy aging sufficiently, we used ordinal logistic regression to test whether arts engagement was associated with the number of domains in which participants were healthy two and four years later (≤1, 2, 3, 4). Third, we also explored whether engagement in receptive and participatory arts activities was associated with each domain of subsequent healthy aging. We used separate logistic regression models to test whether arts engagement at baseline (2014) was associated with chronic diseases, cognitive impairment, physical functioning, and mental health two and four years later.

Finally, we considered that the inclusion of reading in our definition of participatory arts engagement may have influenced our findings, as reading can be classed as a receptive activity and was more common than other participatory activities (Table S12). We therefore repeated our main analyses after removing reading from the measure of participatory arts engagement. For each sensitivity analysis, we generated another 40 imputed data sets with MICE including the additional variables.

## 3 Results

In total, 1269 older adults completed the 2014 CAM and were alive for subsequent HRS waves in 2016 and 2018. Participants’ ages ranged from 50 to 98 years (M = 66.93, SE = 0.28) in 2014 (Table 1). Overall, 62% of participants were women, 42% were retired, 71% were White/Caucasian, 21% were Black/African American, and 8% were of Other races/ethnicities (including American Indian, Alaskan Native, Asian or Pacific Islander, and Hispanic). Most participants had completed high school (52%), were married (57%), and reported living in an area with good to excellent neighborhood safety (89%).

**Table 1:** Demographic characteristics of the sample at baseline, overall and stratified according to the exposures (arts engagement).

	**Overall**	**Participatory arts**				**Receptive arts**		
		0	1	2	3+	Never	<1 a month	≥1 a month
	N (proportion)	Proportion						
Overall engagement	–	18%	32%	29%	22%	34%	42%	24%
Gender								
Women	789 (62%)	46%	61%	65%	73%	60%	64%	62%
Men	480 (38%)	54%	39%	35%	27%	40%	36%	38%
Race/ethnicity								
White/Caucasian	900 (71%)	65%	71%	77%	69%	64%	72%	78%
Black/African American	262 (21%)	22%	22%	17%	23%	26%	21%	14%
Other	107 (8%)	13%	8%	7%	8%	10%	7%	8%
Education								
Less than high school	212 (17%)	33%	17%	14%	8%	32%	9%	8%
High school	659 (52%)	50%	56%	53%	47%	54%	54%	45%
College	256 (20%)	13%	18%	19%	30%	12%	24%	26%
Postgraduate	142 (11%)	4%	9%	14%	16%	3%	13%	21%
Marital status								
Married	723 (57%)	50%	58%	60%	57%	48%	61%	62%
Unmarried	546 (43%)	50%	42%	40%	43%	52%	39%	38%
Employment status								
Employed	466 (37%)	31%	35%	37%	43%	25%	44%	40%
Not working	263 (21%)	26%	21%	20%	18%	29%	16%	17%
Retired	541 (42%)	43%	44%	43%	40%	46%	39%	44%
Neighborhood safety								
Fair/poor	146 (11%)	19%	11%	8%	11%	20%	8%	7%
Excellent/good	1123 (89%)	81%	89%	92%	89%	80%	92%	93%
Mean (SE)								
Age (years)	66.93 (0.28)	68.15 (0.69)	67.17 (0.51)	67.34 (0.50)	65.04 (0.55)	68.95 (0.51)	65.80 (0.41)	66.06 (0.52)
Household income (USD)	74,425 (3176)	47,516 (3977)	78,260 (6709)	75,035 (5380)	90,176 (7354)	38,179 (2033)	89,152 (5768)	100,088 (7446)

*Note.* N = 1269. Results based on 40 multiply imputed data sets.

In 2014, 18% of participants did not engage in any participatory arts (reading, crafts, making music, acting, dancing, writing, needlework, woodwork, and visual arts) in the past 12 months. In contrast, 32% reported doing one of these activities, 29% two activities, and 22% three or more activities. In terms of receptive arts, 34% of participants did not go to a movie, gallery, arts or crafts fair, or live performance in the past 12 months. However, 42% of participants did at least one of these activities less than once a month, and 24% of participants did them once a month or more.

In 2014, 49% of participants were healthy. This dropped to 43% in 2016 and 42% in 2018. Of those who were healthy in 2014, 74% remained healthy in 2016 and 70% remained healthy in 2018. The proportion of the sample classified as healthy on each domain was higher (Table 2). In each domain, healthy aging status was relatively stable. Of the participants free of major chronic diseases in 2014, 85% remained free in 2016 and 2018. Of those with no cognitive impairment in 2014, 89% also had no cognitive impairment in 2016 and 83% in 2018. Of participants with good physical functioning in 2014, 90% also had good functioning in 2016, and 88% in 2018. Finally, 98% of those with good mental health in 2014 also had good mental health in 2016, followed by 96% in 2018.

**Table 2:** Proportion of participants classified as healthy overall and within each domain of healthy aging at each wave.

	**Healthy N (proportion)**		
	2014	2016	2018
Overall	628 (49%)	550 (43%)	527 (42%)
**Domain**			
Chronic diseases	1010 (80%)	912 (72%)	926 (73%)
Cognitive impairment	1013 (80%)	997 (79%)	933 (74%)
Physical functioning	999 (79%)	984 (78%)	936 (74%)
Mental health	1028 (81%)	1022 (81%)	1014 (80%)

*Note.* Total N = 1269 in each year. Results based on 40 multiply imputed data sets, with the domains of healthy aging imputed in a different model to the overall healthy aging variable.

Our sample was similar to the overall HRS 2014 cohort in terms of demographic and socioeconomic characteristics but demonstrated slightly higher levels of healthy aging overall (49% vs 46%; Table S4).

### 3.1 Receptive arts

In the logistic regression models, there was no evidence that doing receptive activities was associated with healthy aging two years later compared to never engaging (Table 3). There was some evidence for an association between receptive engagement and healthy aging four years later before adjustment for covariates. Doing receptive arts once a month or more, compared to never engaging, was associated with 80% higher odds of healthy aging four years later (odds ratio [OR] = 1.80, 95% CI = 1.10 to 2.96, p = .019). However, this evidence was attenuated after adjusting for covariates (adjusted OR [AOR] = 1.44, 95% CI = 0.84 to 2.46, p = .179).

**Table 3:** Logistic regression models testing the associations between receptive and participatory arts engagement (measured in 2014) and subsequent healthy aging (measured in 2016 and 2018).

	**Healthy aging 2 years later**					**Healthy aging 4 years later**				
	Healthy	Unadjusted		Adjusted		Healthy	Unadjusted		Adjusted	
	N (%)	OR (95% CI)	p value	OR (95% CI)	p value	N (%)	OR (95% CI)	p value	OR (95% CI)	p value
**Receptive arts**										
Never	136 (32%)	–		–		126 (29%)	–		–	
<1 a month	267 (50%)	1.34 (0.91, 1.98)	.136	1.00 (0.65, 1.53)	.993	250 (47%)	1.42 (0.94, 2.16)	.098	1.08 (0.67, 1.72)	.758
≥1 a month	147 (49%)	1.33 (0.85, 2.07)	.207	1.04 (0.64, 1.70)	.861	150 (50%)	**1.80 (1.10, 2.96)**	**.019**	1.44 (0.84, 2.46)	.179
**Participatory arts**										
0 activities	80 (35%)	–		–		85 (38%)	–		–	
1 activity	172 (43%)	1.00 (0.62, 1.63)	.985	0.99 (0.58, 1.70)	.981	158 (39%)	0.68 (0.40, 1.16)	.157	0.63 (0.35, 1.12)	.114
2 activities	169 (46%)	1.33 (0.80, 2.22)	.267	1.27 (0.72, 2.24)	.414	154 (42%)	0.83 (0.49, 1.41)	.491	0.74 (0.42, 1.31)	.303
3+ activities	129 (47%)	1.06 (0.63, 1.79)	.813	0.94 (0.52, 1.69)	.835	130 (48%)	0.87 (0.49, 1.54)	.625	0.68 (0.35, 1.30)	.238

*Note.* OR: odds ratio. 95% CI: 95% confidence interval. Total N = 1269. Results based on 40 multiply imputed data sets. Reference categories are indicated with dashes. All models included both receptive and participatory arts exposures and were adjusted for healthy aging status in 2014. Adjusted models were additionally adjusted for age, gender, race/ethnicity, education, marital status, employment status, household income, and neighborhood safety. Bold text indicates p < .05.

### 3.2 Participatory arts

There was no evidence that the number of participatory arts activities engaged in was associated with healthy aging two or four years later, before or after adjusting for covariates (Table 3).

### 3.3 Sensitivity analyses

At baseline, there was evidence for a concurrent association between receptive arts engagement and healthy aging (Table S8). Engaging in receptive arts less than once a month (AOR = 1.48, 95% CI = 1.03 to 2.03, p = .014), or once a month or more (AOR = 1.44, 95% CI = 1.00 to 2.08, p = .050), was associated with higher odds of healthy aging concurrently. However, there was no evidence for an association with participatory arts engagement.

Next, we tested whether arts engagement was associated with the number of domains in which participants were healthy. On average, participants were healthy in 3.2 domains in 2014, 3.1 in 2016, and 3.0 in 2018 (Table S9). There was initially evidence for an association between receptive engagement less than once a month and healthy aging two years later, but this did not survive adjustment for covariates (Table S10). However, there was evidence for an association between receptive engagement and the number of domains in which participants were healthy four years later. Participants who did receptive arts once a month or more had 1.57 times higher odds of being healthy in more than one domain than those who never engaged (AOR = 1.57, 95% CI = 1.04 to 2.37, p = .034). In contrast, there was no evidence for any associations between participatory engagement and healthy aging in any of these models.

We then explored whether arts engagement was associated with each domain of healthy aging separately. In these fully adjusted models, more frequent receptive arts engagement was associated with higher odds of good physical functioning four years later (Table S11). Doing receptive arts less than once a month was associated with 1.57 times higher odds of good physical functioning four years later compared to never engaging (AOR = 1.57, 95% CI = 1.01 to 2.45, p = .047). Moreover, doing receptive arts once a month or more was associated with 2.39 times higher odds of good physical functioning four years later (AOR = 2.39, 95% CI = 1.32 to 4.31, p = .004). However, there was also evidence that engaging in three or more participatory arts activities, compared to doing none, was associated with 52% lower odds of good physical functioning four years later (AOR = 0.48, 95% CI = 0.24 to 0.95, p = .035). There was no evidence that receptive or participatory engagement was associated with any other domains of healthy aging (chronic diseases, cognitive impairment, mental health) or with any of the healthy aging domains two years later.

Finally, we tested whether removing reading from the measure of participatory arts engagement altered our findings. After doing so, 33% of participants did not engage in any participatory arts activities, but 36% did one activity, 20% two activities, and 11% three or more activities. There was still no evidence that the number of participatory arts activities engaged in was associated with healthy aging two or four years later (Table S13).

## 4 Discussion

In this study, frequent engagement in receptive arts activities (going to a movie, gallery, arts or crafts fair, or live performance once a month or more) was associated with increased odds of healthy aging four years later compared to never engaging. However, this evidence was attenuated after adjustment for confounders. In sensitivity analyses, engaging in receptive arts activities once a month or more was associated with higher odds of being healthy in more domains four years later, independent of demographic and socioeconomic confounders and previous healthy aging status. Further sensitivity analyses indicated that this was driven by increased odds of good physical functioning. In contrast, we found no associations between engagement in participatory arts activities (reading, crafts, making music, acting, dancing, writing, needlework, woodwork, and visual arts) and healthy aging. In fact, in sensitivity analyses, there was some indication that doing three or more participatory arts activities, compared to none, was associated with lower odds of good physical functioning four years later. However, this association was not present at two years later or for healthy aging overall. Our findings thus indicate that the associations between art engagement and the maintenance of healthy aging are weak, but might be slightly stronger for receptive than participatory arts engagement.

In older adults, receptive arts engagement has previously been linked to each of the individual domains of healthy aging in isolation (e.g., Bone et al., 2023a; Elsden and Roe, 2020; Fancourt et al., 2020; Groot et al., 2021; Grossi et al., 2012; Ho et al., 2019; Renton et al., 2012; Rogers and Fancourt, 2020). Given this, it was surprising that receptive arts engagement was not more strongly associated with healthy aging. Overall, there was only evidence for a potential benefit of receptive engagement when we considered the number of domains in which participants were healthy four years later. This demonstrates the importance of using a multidimensional definition of healthy aging such that distributed health benefits across multiple domains can have more predictive power. In this study, we adapted a multidimensional definition of healthy aging previously developed in the HRS sample (Kim et al., 2019; Rowe and Kahn, 1997). In contrast to this definition, others define healthy aging as maintaining functional ability to enable wellbeing, regardless of the presence of illness (Tinetti and Fried, 2004; World Health Organization, 2020). In sensitivity analyses, we found an association specifically between receptive engagement and good physical functioning four years later in sensitivity analyses. Physical functioning may be more amenable to change by arts engagement than the other domains of healthy aging. This is promising given that alternative definitions of healthy aging focus on the maintenance of functional ability, independent of existing physical or mental health problems. However, participants in this study were considered to have good physical functioning if they had few difficulties with basic physical activities and activities of daily living. In contrast, the WHO define functional ability more broadly in terms of mobility and ability to meet basic needs, learn grow and make decisions, build and maintain relationships, and contribute (World Health Organization, 2020). Receptive arts engagement is likely to influence all of these aspects of functional ability (Fancourt et al., 2021), but this remains to be explored further.

It is unclear why we did not any find benefits of participatory arts engagement for healthy aging, even concurrently at baseline. Our findings were unexpected given that reviews of participatory arts interventions suggest they can reduce cognitive decline, enhance physical functioning, and improve mental health (Chacur et al., 2022; Christie et al., 2017; National Endowment for the Arts, 2013). However, intervention studies are commonly limited by small, biased samples, attrition, weak statistical analyses (e.g., residual confounding), no replication, and many lack randomization and control groups (National Endowment for the Arts, 2013). It is therefore possible that their conclusions have been overstated. Indeed, similar to our findings, another large observational study did show that receptive engagement, but not participatory engagement, was associated with risk of developing dementia (Fancourt et al., 2020). If this is the case, receptive activities could be more effective at supporting healthy aging as a whole.

If we consider the ‘active ingredients’ of activities measured in this study, the receptive activities measured always involved participants leaving their homes, thereby providing gentle physical activity, which is associated with benefits across all four of the domains of healthy aging included in this study (Durstine et al., 2013; Potter et al., 2011; Sanders et al., 2019; Schuch et al., 2018). In contrast, although some of the participatory activities were active (e.g., dance), the majority could be completed at home seated (e.g., sewing). Indeed, a higher proportion of the sample engaged in sedentary participatory activities than movement-based activities (Table S12). Receptive activities may provide more opportunities for social interactions, community engagement, acts of independence, and novel experiences (Dunphy et al., 2019; Warran et al., 2022), all of which can support healthy aging (Abud et al., 2022). Individual participatory activities could still offer similar benefits but, when exploring the effects of activities as a collective, these ingredients might be more consistently present amongst receptive activities.

A further potential explanation for the lack of association with participatory arts engagement is that differences in measurement affected our ability to detect associations. For receptive activities, we focused on frequency of engagement, whilst for participatory activities we only had counts of the number of different activities engaged in. Someone who dedicated substantial time to one activity was therefore ranked lower than an individual who dedicated small amounts of time to multiple activities, even though their overall time commitment may have been higher. Yet, although activity engagement has traditionally been characterized in terms of frequency of engagement, there is growing evidence that the variety of different activities engaged in is more important for reducing the risk of cognitive decline (Carlson et al., 2012; Jeon et al., 2022), dementia (Moored et al., 2022), and Alzheimer's disease (Friedland et al., 2001) and supporting wellbeing (Jansen et al., 2023). The independent contributions of activity variety and frequency to healthy aging overall, and whether this extends to different forms of arts engagement, requires further investigation (Bielak and Gow, 2023). Additionally, categorizing arts activities as receptive versus participatory is just one approach. Given the heterogeneous nature of activities included within these groupings, it is possible that alternative classifications, different sets of activities, or changes in measurement could have altered the findings. The suggestion that receptive engagement is more strongly associated with healthy aging than participatory engagement is thus difficult to generalize beyond this study and the included activities. Future work could consider individual arts activities as well as alternative categorizations, such as the extent to which activities include social contact, physical activity, and creativity.

Several other factors could also have contributed to the lack of consistent evidence in this study. Any association between arts engagement and healthy aging is likely bidirectional. Older adults may be limited by existing symptoms or functional limitations that preclude doing arts activities, as indicated by the concurrent association between receptive engagement and healthy aging at baseline. Prodromal effects in aging have also been proposed as barriers to arts engagement as their gradual onset cause inactivity (Floud et al., 2021). In adjusted analyses, age accounted for much of the association between arts engagement and healthy aging. Participants had decreasing odds of healthy aging with increasing age independent of previous healthy aging status. Healthy aging status was also relatively stable over time, meaning there was little change to be explained by arts engagement. As the sample was small, we were not able to limit participants to those who were healthy in 2014, and thus could not test whether arts engagement enabled individuals to remain healthy. Late start bias may also have contributed to the overall lack of evidence (Gilsanz et al., 2022); as this study starts at an arbitrary point in participants' lives, any benefits of art engagement may have already been gained, making it difficult to see further change within our relatively short study period. This may have led to more conservative estimates of associations between arts engagement and healthy aging than if the study had started earlier in participants’ lives or included a longer follow-up period.

Additionally, we only measured arts engagement at one time-point, without considering continued or consistent engagement or any new engagement over follow-up, which could also have led to an underestimation of the effects on health. A systematic review concluded that several months of engagement in musical activities might be needed for older adults to receive the maximum benefits (Christie et al., 2017), and observational research has demonstrated that sustained arts engagement has the largest impact on health (Tymoszuk et al., 2019). The arts may be a ‘perishable commodity’ that require consistent engagement to achieve health benefits (Johansson et al., 2001). Considering specific types of arts activities, engagement frequency, and longer-term engagement could be promising avenues for future research.

### 4.1 Strengths and limitations

This study has several strengths. HRS is a large nationally representative cohort of older adults that allowed us to investigate the longitudinal associations between arts engagement and healthy aging, whilst controlling for previous healthy aging status and a range of demographic and socioeconomic confounders. This is particularly important given that socioeconomic factors may explain much of the association between receptive arts engagement and at least one domain of healthy aging (cognition; Fancourt and Steptoe, 2018). Additionally, we used a multidimensional definition of healthy aging that has previously been validated in the wider HRS sample and is robust to the use of varying thresholds across each domain (James et al., 2019; Kim et al., 2019; Rowe and Kahn, 1997; World Health Organization, 2020).

This study also has some limitations. A small random subsample of HRS participants completed the Culture and the Arts Module (CAM), and population weights were not provided to make this subsample representative of the US population. Although this subsample was randomly selected, they were slightly healthier than the core HRS sample at baseline. Those who were healthier or more engaged in the arts could have been more likely to complete the CAM, potentially increasing selection bias, and leading to an overestimation of the association between arts engagement and healthy aging. Despite this, rates of arts engagement were similar to those found in previous research in the US and UK (Bone et al., 2021; Fancourt and Tymoszuk, 2019). Additionally, our sample only included participants who remained alive for subsequent HRS waves in 2016 and 2018. Given the sociodemographic disparities in life expectancy in the US (Arias et al., 2020), this may have led to oversampling of people who were White, of higher socioeconomic position, and healthier, and further contributed to an overestimation of the association between arts engagement and healthy aging. However, classifying participants who were not alive at follow-up as not healthy agers did not alter our findings, indicating instead that our estimates may have been slightly conservative (results available on request). We were limited by the measures included in HRS, meaning that it was not clear whether participatory arts took place in a group, at home alone, or elsewhere. Given that arts engagement may support healthy aging by providing opportunities for social interactions, further research should compare the impacts of activities done alone versus in groups. All measures except cognitive impairment relied on self-report, which may have been subject to biases, limiting the causal inferences that can be made from our findings. Future research should replicate our study using objective measures of art engagement and healthy aging.

Furthermore, women were over-represented in our sample. We recognize that gender is not a binary construct although we had to treat it as such given the way the data was collected. Further, as HRS combined a range of races/ethnicities into the Other race/ethnicity category, we were not able to investigate the influence of race/ethnic identities, and associated racism and cultural caste systems. These limitations underscore the challenges of controlling for demographics and the need for improving methods for measuring and accounting for structural racism in research (Hardeman et al., 2022). By exploring the population-level associations between arts engagement and healthy aging, we conflated individual experiences, so future research should focus on the needs of different groups, who may experience distinctive stressors and outcomes (Williams, 2018). Although we adjusted for a range of covariates, residual confounding is possible. Finally, as the CAM was only included in HRS in 2014, this study included a relatively short follow-up of four years. As more data become available, future research should explore whether any potential benefits of receptive arts engagement for healthy aging are maintained over longer periods.

## 5 Conclusions

In this study, we found limited evidence for associations between receptive or participatory arts engagement and subsequent healthy aging. In sensitivity analyses, there was some evidence that receptive engagement was associated with higher odds of good physical functioning four years later, indicating that it could have some benefits for healthy aging. Although we did not find evidence for an association between participatory arts engagement and healthy aging, the lack of evidence may have been due to the relatively small sample size or limitations in the measure of engagement. Our findings should be confirmed in further research with larger nationally representative samples, longer follow-ups, more detailed exploration of arts engagement, and measures of whether engagement is frequent and sustained. With the unprecedented growth in the number of older adults in the US, identifying ways to ensure older adults remain healthy throughout their extended lifespan is a public health priority. Further research is required to determine whether receptive or participatory arts engagement should be recommended as a community-based intervention to improve the overall health and functioning of older adults.

## Author contributions

JKB, DF, and FB designed the study. MR and JKB conducted the analyses and drafted the manuscript. All authors contributed to the writing, made critical revisions, and approved the final manuscript.

## Funding

The EpiArts Lab, a 10.13039/100000193National Endowment for the Arts Research Lab at the 10.13039/100007698University of Florida, is supported in part by an award from the 10.13039/100000193National Endowment for the Arts (1862896-38-C-20). The opinions expressed are those of the authors and do not represent the views of the National Endowment for the Arts Office of Research & Analysis or the National Endowment for the Arts. The National Endowment for the Arts does not guarantee the accuracy or completeness of the information included in this material and is not responsible for any consequences of its use. The EpiArts Lab is also supported by the 10.13039/100007698University of Florida, the Pabst Steinmetz Foundation, and 10.13039/100015283Bloomberg Philanthropies. DF is also supported by the 10.13039/100010269Wellcome Trust (205407/Z/16/Z).

## Declaration of competing interest

No authors report any conflicts of interest.

## Data Availability

Raw data are available from HRS and the RAND Center for the Study of Aging. Derived data supporting the findings of this study are available from the corresponding author on request.
