# Supplementary material for: Receptive and participatory arts engagement and subsequent healthy aging: Evidence from the Health and Retirement Study
Source: Soc Sci Med. 2023 Oct;334:116198. doi: 10.1016/j.socscimed.2023.116198 (PMC11157693; doi:10.1016/j.socscimed.2023.116198)
Supplement: Multimedia component 1 [file mmc1.docx]

**Supplementary** Materials

**Supplementary** methods

**Table S1.** Illustration of how composite measures were created and combined.

| **Measure** | **Questions included** | **Composite coding** |
| --- | --- | --- |
| Receptive arts engagement | 1. In the past 12 months, did you go to a movie, an art museum or gallery, an arts or crafts fair, or a live performance, such as a concert, play, or reading? *(yes, no)*  2. On average, how often did you go to these types of events in the past twelve months? *(more than once a week, once a week, one to three times a month, less than once a month)* | Never *(q1 = no)*  Less than once a month *(q1 = yes & q2 = less than once a month)*  Once a month or more *(q1 = yes & q2 = one to three times a month, once a week, or more than once a week)* |
| Participatory arts engagement | In the past 12 months did you *(all yes, no):*  1. read novels, short stories, poetry, or plays  2. paint, sculpt, pottery, or ceramics  3. sing or play a musical instrument  4. act in theatre or film  5. dance  6. write stories, poetry, or plays  7. weave, crochet, quilt, needlepoint, knitting, sewing, or jewelry  8. leatherwork, metalwork, or woodwork,  9. photography, graphic design, or filmmaking | None *(no to all qs)*  One activity *(yes to one q)*  Two activities *(yes to two qs)*  Three or more activities *(yes to three or more qs)* |
| Free of chronic diseases | 1. Has a doctor ever told you that you have *(all yes, no):*  cancer or malignant tumor, heart disease, stroke, diabetes, chronic lung disease  2. In the past 2 years, have you received treatment for *(all yes, no): c*ancer or malignant tumor, heart disease, stroke, diabetes, chronic lung disease | Not healthy *(q1 = yes & q2 = yes for one or more disease)*  Healthy *(q1 = no for all diseases, or q2 = no for all diseases)* |
| No cognitive impairment | Sum of scores *(total range from 0 to 27)* on:  1. Immediate free recall *(0-10)*  2. Delayed free recall *(0-10)*  3. Serial sevens subtraction test *(0-5)*  4. Backward count test *(0-2)* | Not healthy *(score of <12)*  Healthy *(score of ≥12)* |
| Good physical functioning | Number of tasks with which participants had difficulties *(yes, no)* from:  1. pushing or pulling large objects  2. lifting or carrying objects weighing ten pounds  3. reaching or extending arms up  4. stooping kneeling or crouching  5. walking across a room  6. dressing  7. eating  8. bathing  9. getting in/out bed  10. using the toilet | Not healthy *(difficulties with <3 activities)*  Healthy *(difficulties with ≥3 activities)* |
| Good mental health | 1. Have you had or has a doctor told you that you have any emotional, nervous, or psychiatric problems? *(yes, no)*  2. Adapted 8-item Center for Epidemiological Studies Depression (CES-D) scale *(total score from 0 to 8)* | Not healthy *(q1 = yes or CES-D total ≥ 4)*  Healthy *(q1 = no & CES-D total < 4)* |
| Overall healthy aging | Composites as defined above:  1. Free of chronic diseases *(yes, no)*  2. No cognitive impairment *(yes, no)*  3. Good physical functioning *(yes, no)*  4. Good mental health *(yes, no)* | Not healthy *(no to any q)*  Healthy *(yes to all qs)* |

**Table S2.** *Correlations between each of the domains of healthy aging at baseline.*

|  | Chronic diseases | Cognitive impairment | Physical functioning |
| --- | --- | --- | --- |
| Cognitive impairment | r = 0.04  (p = .140) |  |  |
| Physical functioning | r = 0.16  (p < .001) | r = 0.23  (p < .001) |  |
| Mental health | r = 0.11  (p < .001) | r = 0.02  (p = .520) | r = 0.23  (p < .001) |

Note. N=1,269. Results based on 40 multiply imputed data sets.

**Table S3.** *Proportion of missing data in imputed variables in the final analytical sample (N=1,269).*

|  | Proportion missing |
| --- | --- |
| Healthy aging 2014 | 6% |
| Healthy aging 2016 | 16% |
| Healthy aging 2018 | 39% |
| Education | 1% |
| Employment status | 1% |
| Neighborhood safety | 2% |

*Note.* There were no missing data in other covariates (gender, race/ethnicity, marital status, age, household income).

**Table S4.** *Characteristics of the included sample at baseline based on 40 multiply imputed data sets (as in Tables 1-2 in the manuscript), compared to the full HRS cohort in 2014.*

|  | Included sample  (n=1,269) | HRS 2014 cohort  (n=18,289) |
| --- | --- | --- |
|  | Proportion | |
| Gender |  |  |
| Women | 62% | 59% |
| Men | 38% | 41% |
| Race/ethnicity |  |  |
| White/Caucasian | 71% | 71% |
| Black/African American | 21% | 20% |
| Other | 8% | 9% |
| Education |  |  |
| Less than high school | 17% | 19% |
| High school | 52% | 53% |
| College | 20% | 19% |
| Postgraduate | 11% | 9% |
| Marital status |  |  |
| Married | 57% | 56% |
| Unmarried | 43% | 44% |
| Employment status |  |  |
| Employed | 37% | 32% |
| Not working | 21% | 21% |
| Retired | 42% | 47% |
| Neighborhood safety |  |  |
| Fair/poor | 11% | 13% |
| Excellent/good | 89% | 87% |
| Classified as healthy: |  |  |
| Overall | 49% | 46% |
| Chronic diseases | 81% | 76% |
| Cognitive impairment | 87% | 78% |
| Physical functioning | 82% | 75% |
| Mental health | 82% | 81% |
|  | Mean (SE) | |
| Age (years) | 66.93 (0.28) | 68.45 (0.08) |
| Household income (USD) | 74,425 (3,176) | 68,970 (1,063) |

*Note.* HRS 2014 cohort defined as those alive and aged 50 or older in 2014 who were interviewed in the wave.

# Complete case analyses

**Table S5.** *Proportion of participants classified as healthy overall and within each domain of healthy aging at each wave in the complete case sample (total N=668 at each wave).*

| Domain | **Healthy N (Proportion)** | | |
| --- | --- | --- | --- |
|  | 2014 | 2016 | 2018 |
| Overall | 355 (53%) | 314 (47%) | 298 (45%) |
| Domain | | | |
| Chronic diseases | 539 (81%) | 483 (72%) | 494 (74%) |
| Cognitive impairment | 581 (87%) | 562 (84%) | 530 (79%) |
| Physical functioning | 549 (82%) | 543 (81%) | 529 (79%) |
| Mental health | 546 (82%) | 548 (82%) | 549 (82%) |

**Table S6.** *Characteristics of the complete case sample at baseline (N=668).*

|  | **Proportion** |
| --- | --- |
| **Participatory arts** |  |
| No activities | 16% |
| 1 activity | 33% |
| 2 activities | 31% |
| 3+ activities | 20% |
| **Receptive arts** |  |
| Never | 30% |
| <1 a month | 44% |
| ≥1 a month | 26% |
| **Gender** |  |
| Women | 58% |
| Men | 42% |
| **Race/ethnicity** |  |
| White/Caucasian | 72% |
| Black/African American | 20% |
| Other | 8% |
| **Education** |  |
| Less than high school | 13% |
| High school | 53% |
| College | 21% |
| Postgraduate | 13% |
| **Marital status** |  |
| Married | 62% |
| Unmarried | 38% |
| **Employment status** |  |
| Employed | 38% |
| Not working | 18% |
| Retired | 44% |
| **Neighborhood safety** |  |
| Fair/poor | 12% |
| Excellent/good | 88% |
|  | **Mean (SD)** |
| Age (years) | 66.14 (9.31) |
| Household income (USD) | 77,549 (100,780) |

**Table S7.** *Logistic regression models testing the associations between receptive and participatory arts engagement (measured in 2014) and subsequent healthy aging (measured in 2016 and 2018) limited to complete cases.*

|  | **Healthy aging 2 years later** | | | | **Healthy aging 4 years later** | | | |
| --- | --- | --- | --- | --- | --- | --- | --- | --- |
|  | Unadjusted | | Adjusted | | Unadjusted | | Adjusted | |
|  | OR (95% CI) | p value | OR (95% CI) | p value | OR (95% CI) | p value | OR (95% CI) | p value |
| **Receptive arts** |  |  |  |  |  |  |  |  |
| <1 a month | 1.55 (0.97, 2.48) | .068 | 1.34 (0.81, 2.22) | .259 | 1.26 (0.80, 1.98) | .329 | 1.06 (0.64, 1.75) | .816 |
| ≥1 a month | 1.63 (0.94, 2.81) | .083 | 1.63 (0.89, 2.99) | .112 | 1.58 (0.92, 1.70) | .095 | 1.54 (0.86, 2.76) | .146 |
| **Participatory arts** |  |  |  |  |  |  |  |  |
| 1 activity | 0.63 (0.35, 1.13) | .118 | 0.61 (0.32, 1.17) | .136 | **0.50 (0.29, 0.86)** | **.013** | **0.49 (0.27, 0.87)** | **.015** |
| 2 activities | 0.95 (0.51, 1.76) | .870 | 0.88 (0.45, 1.73) | .711 | 0.70 (0.40, 1.22) | .207 | 0.64 (0.35, 1.17) | .151 |
| 3+ activities | 0.98 (0.51, 1.88) | .942 | 0.78 (0.38, 1.62) | .512 | 0.97 (0.54, 1.75) | .917 | 0.76 (0.40, 1.44) | .397 |

*Note.* OR: odds ratio. 95% CI: 95% confidence interval. N=668. For both exposures, the reference category was no engagement. All models included both receptive and participatory arts exposures and were adjusted for healthy aging status in 2014. Adjusted models were additionally adjusted for age, gender, race/ethnicity, education, marital status, employment status, household income, and neighborhood safety. Bold text indicates p < .05.

# Concurrent associations at baseline

**Table S8.** *Logistic regression models testing the associations between receptive and participatory arts engagement (measured in 2014) and concurrent healthy aging (measured in 2014).*

|  | **Healthy aging concurrently (2014)** | | | |
| --- | --- | --- | --- | --- |
|  | Unadjusted | | Adjusted | |
|  | OR (95% CI) | p value | OR (95% CI) | p value |
| **Receptive arts** |  |  |  |  |
| <1 a month | **2.37 (1.80, 3.12)** | **<.001** | **1.48 (1.08, 2.03)** | **.014** |
| ≥1 a month | **2.26 (1.64, 3.12)** | **<.001** | **1.44 (1.00, 2.08)** | **.050** |
| **Participatory arts** |  |  |  |  |
| 1 activity | 1.33 (0.94, 1.87) | .107 | 1.15 (0.80, 1.65) | .456 |
| 2 activities | 1.08 (0.75, 1.54) | .684 | 0.90 (0.62, 1.32) | .597 |
| 3+ activities | 1.28 (0.87, 1.87) | .205 | 0.99 (0.66, 1.51) | .978 |

*Note.* OR: odds ratio. 95% CI: 95% confidence interval. N=1,269. Results based on 40 multiply imputed data sets. For both exposures, the reference category was no engagement. All models included both receptive and participatory arts exposures. Adjusted models were adjusted for age, gender, race/ethnicity, education, marital status, employment status, household income, and neighborhood safety. Bold text indicates p < .05.

# Number of domains in which participants were healthy

**Table S9.** *Distribution of the number of domains in which participants were healthy in each wave.*

| Number of domains classified as healthy | **Proportion** | | |
| --- | --- | --- | --- |
|  | 2014 | 2016 | 2018 |
| 0-1 | 7% | 9% | 10% |
| 2 | 14% | 16% | 17% |
| 3 | 29% | 33% | 32% |
| 4 | 49% | 43% | 41% |

*Note.* Total N=1,269.

**Table S10.** *Ordinal logistic regression models testing the associations between receptive and participatory arts engagement (measured in 2014) and the number of domains in which participants were healthy (measured in 2016 and 2018).*

|  | **Healthy aging 2 years later** | | | | **Healthy aging 4 years later** | | | |
| --- | --- | --- | --- | --- | --- | --- | --- | --- |
|  | Unadjusted | | Adjusted | | Unadjusted | | Adjusted | |
|  | OR (95% CI) | p value | OR (95% CI) | p value | OR (95% CI) | p value | OR (95% CI) | p value |
| **Receptive arts** |  |  |  |  |  |  |  |  |
| <1 a month | **1.38 (1.01, 1.87)** | **.041** | 1.06 (0.76, 1.47) | .743 | **1.48 (1.04, 2.10)** | **.030** | 1.17 (0.80, 1.71) | .430 |
| ≥1 a month | 1.41 (0.97, 2.04) | .069 | 1.10 (0.73, 1.64) | .653 | **1.95 (1.33, 2.84)** | **.001** | **1.57 (1.04, 2.37)** | **.034** |
| **Participatory arts** |  |  |  |  |  |  |  |  |
| 1 activity | 0.90 (0.61, 1.33) | .600 | 0.83 (0.55, 1.25) | .368 | 0.70 (0.45, 1.08) | .103 | 0.65 (0.42, 1.03) | .068 |
| 2 activities | 1.08 (0.71, 1.63) | .728 | 0.99 (0.64, 1.53) | .959 | 0.87 (0.54, 1.39) | .551 | 0.81 (0.49, 1.34) | .406 |
| 3+ activities | 0.87 (0.57, 1.33) | .514 | 0.76 (0.48, 1.19) | .229 | 0.76 (0.47, 1.24) | .274 | 0.67 (0.40, 1.13) | .130 |

*Note.* OR: odds ratio. 95% CI: 95% confidence interval. N=1,269. Results based on 40 multiply imputed data sets. For both exposures, the reference category was no engagement. All models included both receptive and participatory arts exposures and were adjusted for healthy aging status in 2014. Adjusted models were additionally adjusted for age, gender, race/ethnicity, education, marital status, employment status, household income, and neighborhood safety. Bold text indicates p < .05.

# Healthy aging domains separately

**Table S11.** *Logistic regression models testing the associations between receptive and participatory arts engagement (measured in 2014) and subsequent healthy aging within each of the four domains (measured in 2016 and 2018).*

|  | **Chronic diseases** | | **Cognitive impairment** | | **Physical functioning** | | **Mental health** | |
| --- | --- | --- | --- | --- | --- | --- | --- | --- |
|  | OR (95% CI) | p value | OR (95% CI) | p value | OR (95% CI) | p value | OR (95% CI) | p value |
| **Outcomes 2 years later** | | | | | | | | |
| **Receptive arts** |  |  |  |  |  |  |  |  |
| <1 a month | 0.95 (0.62, 1.46) | .821 | 1.44 (0.94, 2.22) | .097 | 1.16 (0.75, 1.81) | .499 | 0.79 (0.37, 1.71) | .556 |
| ≥1 a month | 0.81 (0.50, 1.30) | .380 | 1.34 (0.80, 2.23) | .267 | 1.64 (0.93, 2.91) | .089 | 1.78 (0.67, 4.71) | .248 |
| **Participatory arts** |  |  |  |  |  |  |  |  |
| 1 activity | 0.76 (0.47, 1.24) | .272 | 1.34 (0.79, 2.25) | .274 | 0.89 (0.53, 1.50) | .661 | 2.59 (0.80, 8.36) | .111 |
| 2 activities | 1.04 (0.62, 1.74) | .894 | 1.58 (0.89, 2.79) | .115 | 1.14 (0.66, 1.99) | .638 | 2.50 (0.89, 7.06) | .083 |
| 3+ activities | 0.93 (0.52, 1.66) | .814 | 0.91 (0.50, 1.63) | .743 | 1.01 (0.54, 1.90) | .964 | 1.79 (0.56, 5.78) | .327 |
| **Outcomes 4 years later** | | | | | | | | |
| **Receptive arts** |  |  |  |  |  |  |  |  |
| <1 a month | 0.97 (0.63, 1.50) | .888 | 1.45 (0.93, 2.24) | .098 | **1.57 (1.01, 2.45)** | **.047** | 1.03 (0.50, 2.14) | .916 |
| ≥1 a month | 1.18 (0.72, 1.94) | .511 | 1.40 (0.83, 2.37) | .209 | **2.39 (1.32, 4.31)** | **.004** | 1.22 (0.56, 2.68) | .605 |
| **Participatory arts** |  |  |  |  |  |  |  |  |
| 1 activity | 0.87 (0.54, 1.41) | .567 | 1.10 (0.62, 1.92) | .751 | 0.61 (0.34, 1.09) | .092 | 1.49 (0.63, 3.53) | .361 |
| 2 activities | 0.99 (0.59, 1.66) | .955 | 1.12 (0.64, 1.96) | .684 | 0.74 (0.41, 1.34) | .318 | 1.21 (0.52, 2.81) | .650 |
| 3+ activities | 1.13 (0.63, 2.06) | .679 | 0.78 (0.43, 1.44) | .434 | **0.48 (0.24, 0.95)** | **.035** | 1.34 (0.48, 3.73) | .572 |

*Note.* OR: odds ratio. 95% CI: 95% confidence interval. N=1,269. Results based on 40 multiply imputed data sets. All outcomes were coded 0,1 such that 1 indicated healthy aging. An odds ratio larger than 1 thus means that arts engagement is associated with lower risk of chronic diseases and cognitive impairment or better physical functioning and mental health. For both exposures, the reference category was no engagement. All models included both receptive and participatory arts exposures and were adjusted for the outcome in 2014, age, gender, race/ethnicity, education, marital status, employment status, household income, and neighborhood safety. Bold text indicates p<.05.

# Excluding reading from participatory arts

**Table S12.** *Breakdown of participatory arts engagement by activity type.*

| **Activity** | **Proportion of sample engaged** |
| --- | --- |
| Read novels, short stories, poetry, or plays | 54% |
| Paint, sculpt, pottery, or ceramics | 8% |
| Sing or play a musical instrument | 20% |
| Act in theatre or film | 1% |
| Dance | 28% |
| Write stories, poetry, or plays | 7% |
| Weave, crochet, quilt, needlepoint, knitting, sewing, or jewelry | 28% |
| Leatherwork, metalwork, or woodwork | 12% |
| Photography, graphic design, or filmmaking | 11% |

**Table S13.** *Logistic regression models testing the associations between receptive and participatory arts engagement (measured in 2014) and subsequent healthy aging (measured in 2016 and 2018) after excluding reading from the definition of participatory engagement.*

|  | **Healthy aging 2 years later** | | | | **Healthy aging 4 years later** | | | |
| --- | --- | --- | --- | --- | --- | --- | --- | --- |
|  | Unadjusted | | Adjusted | | Unadjusted | | Adjusted | |
|  | OR (95% CI) | p value | OR (95% CI) | p value | OR (95% CI) | p value | OR (95% CI) | p value |
| **Receptive arts** |  |  |  |  |  |  |  |  |
| <1 a month | 1.34 (0.92, 1.94) | .125 | 0.99 (0.66, 1.49) | .968 | 1.45 (0.96, 2.19) | .079 | 1.09 (0.68, 1.74) | .721 |
| ≥1 a month | 1.37 (0.89, 2.12) | .157 | 1.05 (0.65, 1.70) | .828 | **1.81 (1.16, 2.84)** | **.010** | 1.42 (0.88, 2.28) | .150 |
| **Participatory arts** |  |  |  |  |  |  |  |  |
| 1 activity | 1.07 (0.75, 1.53) | .710 | 1.06 (0.73, 1.53) | .766 | 1.01 (0.68, 1.51) | .946 | 0.99 (0.65, 1.50) | .946 |
| 2 activities | 1.18 (0.76, 1.84) | .463 | 1.04 (0.66, 1.64) | .866 | 0.78 (0.47, 1.28) | .328 | 0.65 (0.38, 1.11) | .113 |
| 3+ activities | 0.93 (0.54, 1.61) | .801 | 0.84 (0.48, 1.47) | .540 | 1.04 (0.59, 1.83) | .893 | 0.87 (0.48, 1.59) | .655 |

*Note.* OR: odds ratio. 95% CI: 95% confidence interval. N=1,269. Results based on 40 multiply imputed data sets. For both exposures, the reference category was no engagement. All models included both receptive and participatory arts exposures and were adjusted for healthy aging status in 2014. Adjusted models were additionally adjusted for age, gender, race/ethnicity, education, marital status, employment status, household income, and neighborhood safety. Bold text indicates p < .05.
